# Identification of a Small Molecule That Inhibits the Interaction of LPS Transporters LptA and LptC

**DOI:** 10.3390/antibiotics11101385

**Published:** 2022-10-10

**Authors:** Xiaowei Dai, Min Yuan, Yu Lu, Xiaohong Zhu, Chao Liu, Yifan Zheng, Shuyi Si, Lijie Yuan, Jing Zhang, Yan Li

**Affiliations:** 1 Key Laboratory of Antimicrobial Agents, Institute of Medicinal Biotechnology, Chinese Academy of Medical Sciences and Peking Union Medical College, Beijing 100050, China; 2 Hebei Key Laboratory for Chronic Diseases, Tangshan Key Laboratory for Preclinical and Basic Research on Chronic Diseases, School of Basic Medical Sciences, North China University of Science and Technology, Tangshan 063210, China; 3 State Key Laboratory for Infectious Diseases Prevention and Control, Collaborative Innovation Center for Diagnosis and Treatment of Infectious Disease, National Institute for Communicable Disease Control and Prevention, Chinese Center for Disease Control and Prevention, Beijing 102206, China

**Keywords:** lipopolysaccharide, lipopolysaccharide transport (Lpt) protein, *Escherichia coli*, LptA/LptC interaction, yeast two-hybrid, antibacterial agent

## Abstract

The need for novel antibiotics has become imperative with the increasing prevalence of antibiotic resistance in Gram-negative bacteria in clinics. Acting as a permeability barrier, lipopolysaccharide (LPS) protects Gram-negative bacteria against drugs. LPS is synthesized in cells and transported to the outer membrane (OM) via seven lipopolysaccharide transport (Lpt) proteins (LptA–LptG). Of these seven Lpt proteins, LptC interacts with LptA to transfer LPS from the inner membrane (IM) to the OM, and assembly is aided by LptD/LptE. This interaction among the Lpt proteins is important for the biosynthesis of LPS; therefore, the Lpt proteins, which are significant in the assembly process of LPS, can be a potential target for new antibiotics. In this study, a yeast two-hybrid (Y2H) system was used to screen compounds that could block LPS transport by inhibiting LptA/LptC interaction, which finally disrupts the biosynthesis of the OM. We selected the compound IMB-0042 for this study. Our results suggest that IMB-0042 disrupts LptA/LptC interaction by binding to both LptA and LptC. *Escherichia coli* cells, when treated with IMB-0042, showed filament morphology, impaired OM integrity, and an accumulation of LPS in the periplasm. IMB-0042 inhibited the growth of Gram-negative bacteria and showed synergistic sensitization to other antibiotics, with low cytotoxicity. Thus, we successfully identified a potential antibacterial agent by using a Y2H system, which blocks the transport of LPS by targeting LptA/LptC interaction in *Escherichia coli*.

## 1. Introduction

The increasing prevalence of antibiotic resistance in clinically relevant pathogens, especially Gram-negative bacteria, has caused a global public health concern. Despite increasing awareness, the existing antibacterial drugs have limitations that have necessitated the development of new therapeutic strategies [1]. The cell envelope of Gram-negative bacteria consists of an inner membrane (IM), a thin layer of peptidoglycan, an outer membrane (OM), and a periplasmic space between IM and OM [2]. The OM is an asymmetric bilayer with phospholipids (PLs) and lipopolysaccharides (LPS). As a layer of tightly packed molecules, LPS is the permeability barrier for Gram-negative strains, which protects the bacteria from harmful hydrophobic compounds, such as antibiotics [3,4]. The protective role of LPS in Gram-negative bacteria makes the assembly process of LPS a potential target for the development of new drugs [5].

**Figure 1:**
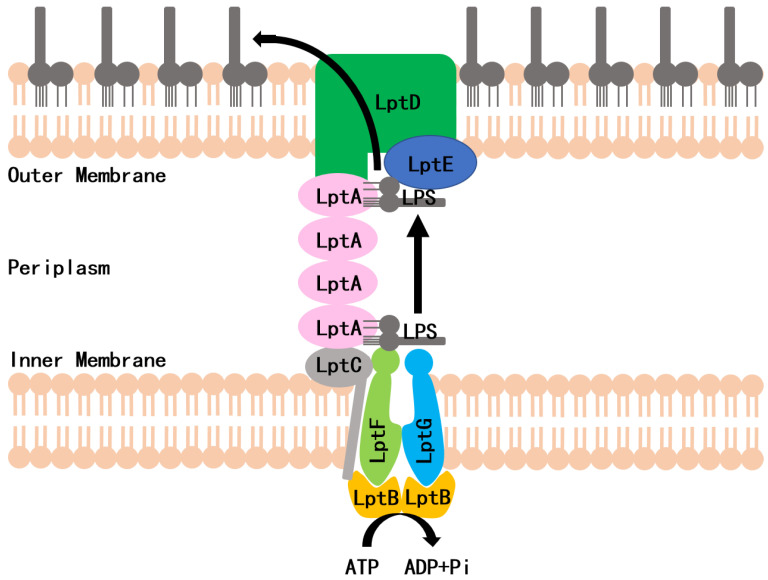
Schematic of the lipopolysaccharide transportation (Lpt) system.

LPS is synthesized in the cytoplasm and asymmetrically distributed to the OM by the LPS transport (Lpt) system, which is the molecular machinery that transports and assembles LPS. In *Escherichia coli*, the Lpt machinery is composed of seven essential proteins (LptA to LptG) that span the entire cell envelope (Figure 1) [6,7,8]. The LptB_2_FG complex functions as an ATP-binding cassette (ABC) transporter and is responsible for the extraction of LPS from the IM [9]. The bitopic protein LptC binds to the LptB_2_FG complex at the outer leaflet of the IM and serves as the docking site for LptA [9]. LptA accepts LPS through its interaction with LptC and transports LPS to the OM. The LptDE complex, located on the OM, then translocates LPS across the OM for LPS assembly at the cell surface [10,11]. Lpt protein depletion in conditional expression mutants or mutations that interfere with protein–protein interactions can inhibit the formation of the bridge, which results in the accumulation of newly synthesized LPS in the IM and the formation of membranous bodies in the periplasm leading to cell growth arrest [12,13,14]. The Lpt complex is a promising target for designing new antibiotics against Gram-negative bacteria. Very recently, a screening strategy, based on the Bacterial Adenylate Cyclase Two-Hybrid (BACTH) system, was used to thoroughly investigate the in vivo effect of the antimicrobial peptide, thanatin, that perturbs the LptA/LptC interaction. In an earlier study, we used a yeast two-hybrid (Y2H) system to screen the LptA/LptC interaction inhibitor, and IMB-881 was selected as a compound that could successfully inhibit the growth of *E. coli* cells by disrupting LptA/LptC interaction [15]. Here, we applied this Y2H model for further screening and found another inhibitor, IMB-0042, which blocks the LptA/LptC interaction and shows potent antibacterial activity with low toxic effects.

## 2. Materials and Methods

### 2.1. Strains and Compound Library

AH109(pAD-LptA+pBD-LptC),AH109(pAD-T+pBD-53), *pET-16a-lptA*-BL21 and *pET-16a-lptC*-BL21 were obtained from the Institute of Medicinal Biotechnology laboratory [15]. Strain and vector information are provided in the supplementary data (Table S1). The library of substances that were used for screening contains both natural products (from the Institute of Medicinal Biotechnology) and synthetic compounds (from Enamine). A total of 100,000 compounds were screened.

### 2.2. Compound Library Screen

AH109(pAD-LptA+pBD-LptC) was used to screen compounds that affected LptA/LptC interaction and AH109(pAD-T+pBD-53) was used as the negative control. The screening assays were performed as described elsewhere, with minor modifications [15]. In brief, AH109 (pAD-LptA+pBD-LptC) (OD_600_ = 0.6) were diluted 100-fold in SD/-Leu-Trp-Ade-His, 198 µL dilution, and 2 µL compound were added to each well. The final concentration of each compound was 100 µg/mL in 1% DMSO. Yeast cells were incubated at 30 ℃ for 48 h and the compounds inhibiting the growth of AH109(pAD-LptA+pBD-LptC) were selected. Then, the minimum inhibitory concentration (MIC), the lowest drug concentration that inhibits cell growth of these compounds to inhibit the growth of AH109(pAD-LptA+pBD-LptC) and AH109 (pAD-T+pBD-53), was detected. The compounds exhibiting lower toxicity (two or more multiples of MIC) toward the growth of AH109 (pAD-LptA+pBD-LptC) were selected.

### 2.3. Quantitative β-Galactosidase (β-Gal) Assay

The primary selected compounds were further assessed using a quantitative β-gal assay. In this assay, AH109(pAD-LptA+pBD-LptC) and AH109 (pAD-T+pBD-53) cells treated with DMSO or IMB-0042 in SD/-Ade-His medium were harvested by centrifugation (8000 rpm, 5 min) and the β-gal activity was detected with a yeast β-galactosidase activity assay kit (GENMED). The β-gal activity was carried out according to the following calculation: 1000 × A_420_/(t × V × OD_600_). In this calculation, t is the incubation time (min) and V is the volume of cell cultures used for the assay (mL).

### 2.4. Expression and Purification of Recombinant Lpt Proteins

*lptA*(GenBank, ID:QBC14848.1) and *lptC*(GenBank, ID:QBC14847.1) were synthesized with *Xho* I and *BamH* restriction sites. The gene sequence and optimization of GC are shown in Figure S1. The *pET-28a-lptA* and *pET-28a*-*lptC* were constructed and transferred into *E. coli* BL21(DE3) cells to express His-tagged LptA and LptC. The expression of the fusion proteins was induced by adding 0.5 mM IPTG into the cell culture and grown overnight at 30 °C. The expression of protein was determined by 12% SDS-PAGE, followed by coomassie blue staining. Cells were collected by centrifugation (8000× *g*) for 10 min at 4 °C and then suspended in lysis buffer (provided by Constant Systems Limited, UK). The lysed cell solution was added to the pre-treated His-Trap HP column (GE Healthcare) for 60 min at 4 °C. LptA and LptC proteins were collected by elution buffer. The purified protein was verified by western blotting, using an anti-His antibody (Com Win Biotech Co., Beijing, China) and an HRP-labeled secondary antibody. Protein concentration was determined by the Bradford method.

### 2.5. Surface Plasmon Resonance (SPR) Analysis

The inhibition of the LptA/LptC interaction by IMB-0042 was detected by SPR in vitro, using the Reichert SPR system as previously described [16]. The interaction between the proteins and the compound IMB-0042 was measured using a Carboxymethyl Dextran sensor chip in PBST running buffer (0.01 M Na Phosphate, 0.15 M NaCl, 0.05% Tween 20). Proteins LptA or LptC were injected onto the surface of the chip at a rate of 10 µL/min and the channel-unloaded protein was taken as the reference. The compound IMB-0042 was diluted in running buffer and passed over the chip at a flow rate of 25 µL/min. The amount of the loading proteins should be approximately 2000RU. Initially, LptC was immobilized on a mixed, self-assembled monolayer (SAM) sensor chip (Reichert Technologies). LptA protein, in a PBST buffer, was passed over the SAM sensor chip to detect the interaction between LptA and LptC. The sensor surface was regenerated after each association and dissociation cycle by injecting 1 M NaCl for 90 s. LptA (30 µg/mL) was mixed with varying concentrations of the compound IMB-0042 (in PBST) in equal volumes so that the final concentration of LptA was 15 ug/mL and the compounds were 0 ug/mL, 1 ug/mL, 2 ug/mL, and 4 ug/mL. The protein-compound mixture passed over the SAM sensor chip to allow binding with LptC protein and to detect any inhibition via the sensor. Both association (k_on_) and dissociation (k_off_) constant values were determined with TraceDrawer 1.7.1. KD = Koff/Kon.

### 2.6. The Inhibition of E. coli Growth by the Compound IMB-0042

The growth inhibition of *E. coli* ATCC 25922 by IMB-0042 was determined in a 96-well plates, following the guidelines of the Clinical and Laboratory Standards Institute (CLSI). The logarithmic phase cells were diluted to 10^5^ CFU/mL in LB medium, and a series of different concentrations of IMB-0042 (ranging from 1.56 µg/mL to 100 µg/mL) were added separately.

Different concentrations of IMB-0042 (ranging from 3.125 µg/mL to 50 µg/mL ) were added to the tubes containing the logarithmic phase cells (diluted to 10^5^ CFU/mL in LB medium), and the cells treated with 0.5% DMSO were used as control. The cells were then incubated at 37 °C with shaking at 150 rpm/min, and samples were collected every hour to measure optical density at 600 nm (OD_600_) to plot the bacterial growth curve.

A checkerboard assay was used to detect the synergistic effect of IMB-0042 with other antibiotics against the *E. coli* strain (ATCC 25922) as CLSI. The fractional inhibitory concentration index (FICI) was calculated according to the following formula:

FICI = (MIC_antibiotics combination_)/(MIC_antibiotics alone_) + (MIC_IMB-0042 combination_)/(MIC_IMB-0042 alone_). FICI ≤ 0.5 was considered as synergistic effect.

### 2.7. Testing the Mode of Action: Bacteriostatic vs. Bactericidal Mode

Cell cultures of *E. coli* ATCC 25922 in log-phase were diluted to 2 × 10^5^ CFU/mL and cultured in LB medium containing IMB-0042 (from 6.25 µg/mL to 100 µg/mL) or 0.5% DMSO with shaking at 150 rpm/min. Bacteria were collected every hour and spread onto LB plates after serial dilution. The plates were incubated at 37 °C for 24 h and the number of colonies was counted.

### 2.8. Light Microscopy and Transmission Electron Microscopy (TEM)

IMB-0042 (concentrations ranging from 1/8 MIC to 1/4 MIC ) or DMSO (0.1%) was added to the culture of *E. coli* ATCC 25922, which was diluted to 5 × 10^6^ CFU/mL in LB medium. The cells were collected every hour. The crystal violet-stained bacterial smears were prepared, and slides were examined under a microscope. The images were obtained through a ZEISS A-Plan 100×/1.25 oil immersion and analyzed with AxioVision Rel.4.8. Other samples were fixed first with 2.5% glutaraldehyde and then with 1% osmium tetroxide in sodium cacodylate buffer for 2 h. The samples were then dehydrated in increasing concentrations of ethanol and infiltrated with Araldite resin. Embedded samples were sectioned and observed at 80 kV on a JEM-1400 TEM (HT7800, 6-6, Marunouchi 1-chome, Chiyoda-ku, Tokyo, Japan).

### 2.9. In Vitro Accumulation of Ethidium Bromide (EtBr)

*E. coli* ATCC 25922 cells were diluted to 5 × 10^6^ CFU/mL in LB medium, treated either with IMB-0042 (concentrations ranging from 1/8 MIC to MIC) or DMSO (0.1%) and grown for 12 h. All the samples were adjusted to have a similar cell number (OD_600_ = 0.2) and poured on the black microtiter plates with clear bottoms (100 µL in each well). The ethidium bromide (EtBr) was then added to each well to make a final concentration of 4 µg/mL. Fluorescence intensity was recorded every 60 s for 10 min using a fluorescence plate reader (EnSpire R2300, Perkin Elmer, Waltham, MA, USA). The emission and excitation wavelengths were 530 nm and 600 nm, respectively.

### 2.10. Cytotoxicity Assay

The MTT method was used to detect the cytotoxic effect of IMB-0042 on the 293T cells. The 293T cells were diluted to a concentration of 5 × 10^4^ cells/mL in DMEM medium containing 2% fetal bovine serum and added to 96-well plates. The logarithmic cells were then incubated in a DMEM medium containing different concentrations of IMB-0042 (ranging from 0.78 µg/mL to 50 µg/mL) for 48 h. MTT reagent was added to this culture and incubated for another 4 h. Absorbance was measured at 570 nm following the mixture of DMSO (50 µL). IC_50_ values were calculated from the concentration-response curve.

### 2.11. Statistical Analysis

All data analyses were performed using GraphPad Prism 8. The *t*-test was used to determine the variations between the treatment and the control groups. A *p*-value < 0.05 (two-tailed) was considered statistically significant. The data were presented as mean ± SD values.

## 3. Results

### 3.1. Compound IMB-0042 Inhibits LptA/LptC Interaction in the Y2H System

Lpt complex is a promising target for designing new antibiotics against Gram-negative bacteria. In a previous study, a yeast two-hybrid system (Y2H) was used to screen the inhibitor of the LptA/LptC interaction and IMB-881 was selected from 200,000 compounds [15]. In this Y2H model, the plasmids pAD-LptA and pBD-LptC were co-transformed into AH109 cells and the interaction of LptA/LptC bring the BD and AD of transcription factor Gal4 together and activate the transcription of three reporter genes *ade,* *his, and lacZ* in AH109 cells, which can be validated by detecting the growth on SD/-Leu-Trp-Ade-His medium and β-gal activity. If a compound inhibits the interaction of LptA/LptC, the growth of AH109 (pAD-LptA+pBD-LptC) on SD/-Leu-Trp-Ade-His medium will be inhibited. AH109 yeast cells with plasmids pAD-T and pBD-53 were used as control to exclude the compound, which disrupts the Gal4 expression system or shows antifungal activity. AH109 (pAD-T+pBD-53) grow well on a SD/-Leu-Trp-Ade-His medium and show strong β-gal activity because of the interaction of expressed proteins p53 and SV40-T [17].

**Figure 2:**
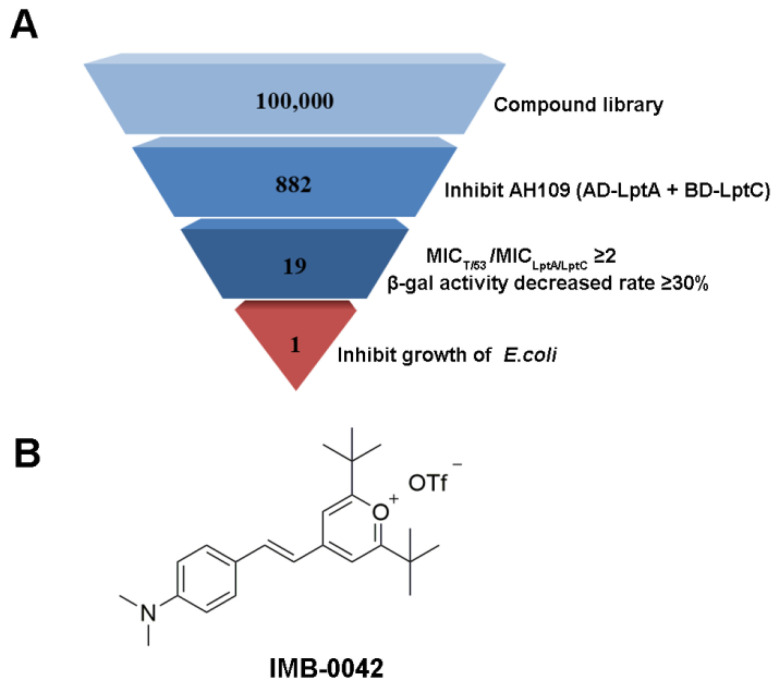
Summary of inhibitor screen. (**A**) In total, 100,000 compounds were screened for inhibitor of LptA/LptC interaction; 882 compounds showed inhibitory activity against AH109 (pAD-LptA+pBD-LptC) in the primary screen, whereas secondary screening verified 19 compounds as hits. Among them, only compound IMB-0042 showed inhibitory activity against *E. coli* ATCC 25922 at 50 µg/mL. (**B**) Structure of the compound IMB-0042.

Here, we applied this Y2H model for further high-throughput screening for 100,000 compounds. To reduce the burden of screening, only AH109 (pAD-LptA+pBD-LptC) was used in the preliminary stages, and the compounds that inhibited the growth of AH109 (pAD-LptA+pBD-LptC) on SD/-Leu-Trp-Ade-His medium at 100 µg/mL were selected. We further evaluated the MICs of the primarily selected compounds against AH109 (pAD-LptA+pBD-LptC) and AH109 (pAD-T+pBD-53). The ones that exhibited lower toxicity (two or more multiples of MIC) toward AH109 (pAD-LptA+pBD-LptC), compared with AH109 (pAD-T+pBD-53), were chosen. Then, the effects of these compounds (at MIC concentration) were tested on the expression of *lacZ* by estimating the β-gal enzyme activity in AH109 (pAD-LptA+pBD-LptC). The compounds that reduced the β-gal enzyme activity by more than 30% were selected. Finally, the compounds among those that were already selected, that inhibited the growth of *E. coli* (MICs were less than or equal to 100 µg/mL) were selected. The screening result is shown in Figure 2. The compound, IMB-0042, was the selected candidate for exhibiting high inhibitory effects.

**Figure 3:**
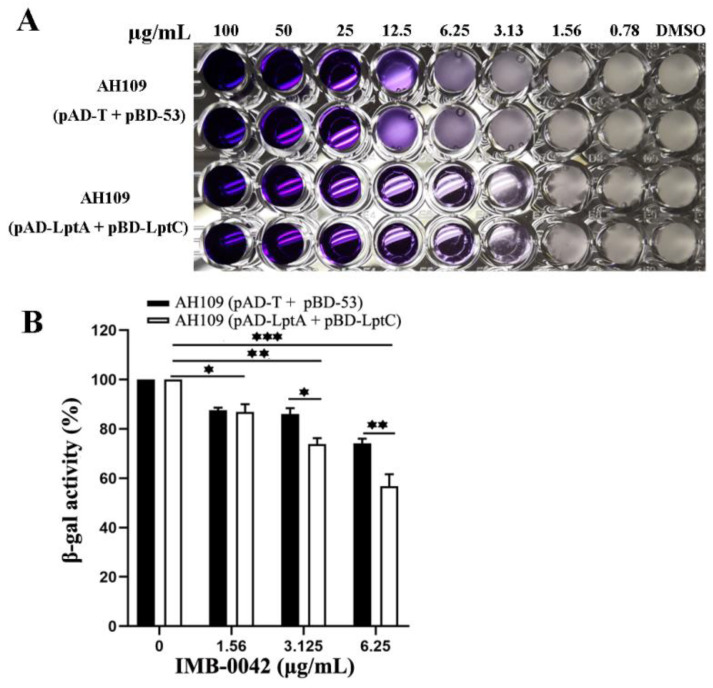
IMB-0042 disrupts the LptA/LptC interaction in Y2H system. (**A**) Growth inhibition of yeast cells by IMB-0042. Yeast AH109 strains with indicated plasmids were inoculated into SD/-Leu-Trp-Ade-His dropout medium in 96-well plates with duplicates. The final concentration of IMB-0042 ranged from 0.78 µg/mL to 100 µg/mL, and their growth was examined after 48 h incubation at 30 °C. (**B**) The inhibition of β-gal activity in AH109 (pAD-LptA+pBD-LptC) cells by IMB-0042 at different concentrations (from 1.56 µg/mL to 6.25 µg/mL) in SD/-Ade-His dropout medium. Strain AH109 (pAD-T+pBD-53) was used as the control. The values represent the percentage of β-gal activity in cells treated with IMB-0042 over that of untreated cells. The results shown here are the average units from triplicated assays. * *p* < 0.05, ** *p* < 0.01, and *** *p* < 0.001.

The MIC of IMB-0042 against AH109 (pAD-LptA+pBD-LptC) cells was 3.125 µg/mL, but for AH109 (pAD-T+pBD-53), it was 25 µg/mL (Figure 3A). In AH109 (pAD-LptA+pBD-LptC) cells, β-gal activity decreased with the increase in IMB-0042 concentration, but in AH109 (pAD-T+pBD-53) cells, this reduction was lower than that observed in AH109 (pAD-LptA+pBD-LptC) (Figure 3B). These results indicated that compound IMB-0042 could specifically inhibit the LptA/LptC interaction.

### 3.2. Compound IMB-0042 Inhibits LptA/LptC Interaction In Vitro

**Figure 4:**
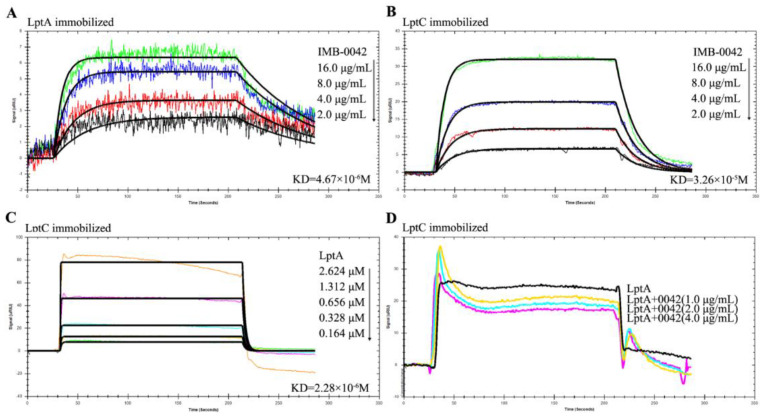
SPR analysis for the binding of compound IMB-0042 with LptA and LptC proteins and the effect of IMB-0042 on LptA/LptC interaction. (**A**,**B**) The binding of compound IMB-0042 to LptA or LptC protein. Serially concentrated solutions of IMB-0042 (from 2.0 µg/mL to 16 µg/mL) were injected into the chamber with a Carboxymethyl Dextran sensor chip coated with LptA (**A**) or LptC. (**B**) The change of response units is shown. (**C**) Demonstration of LptA/LptC interaction. LptC was immobilized on the SAM sensor chip, while LptA, with concentrations ranging from 0.164 µM to 2.624 µM, was passed through the chip. The change in response units is shown. (**D**) Compound IMB-0042 blocked LptA/LptC interaction. LptA proteins preincubated with or without compound IMB-0042 were injected into a chamber with an LptC-coated SAM sensor chip. The change of response units was measured over time.

The blocking effect of IMB-0042 on LptA/LptC was further analyzed by SPR technology, which is widely used in the quantitative determination of intermolecular interactions [18]. We used *E. coli* to express the LptA and LptC proteins with a 6-His tag at the N-terminal, purified the proteins by AKTA system, and detected the interaction with SPR. The detection of expression and purified proteins is shown in Figure S1. To confirm whether compound IMB-0042 could bind to LptA or LptC, both proteins were immobilized on a Carboxymethyl Dextran sensor chip, and then IMB-0042 (at different concentrations) was allowed to flow through the chip. The loading amount of LptA protein was 2257 response units (RUs) and the loading amount of LptC protein was 2420 RUs. The SPR analysis showed that IMB-0042 could bind to both LptA and LptC proteins (Figure 4A,B, Table 1). Then, LptC was immobilized on a SAM sensor chip and LptA was passed over the chip to detect the interaction between LptA and LptC. Measurable changes in the response units indicated that LptC could bind to LptA in a dose-dependent manner. The kinetic rate constant, KD, was determined to be 2.28 × 10^−6^ (Figure 4C, Table 1). To detect the effect of IMB-0042 on LptA/LptC interaction, LptC (800 RU) was immobilized on the surface of SAM sensor chips; meanwhile, LptA and IMB-0042 (in different concentrations) were passed over the chip. As shown in Figure 4D, the binding signal values were reduced in a concentration gradient manner.

### 3.3. Antibacterial Activity of Compound IMB-0042

**Figure 5:**
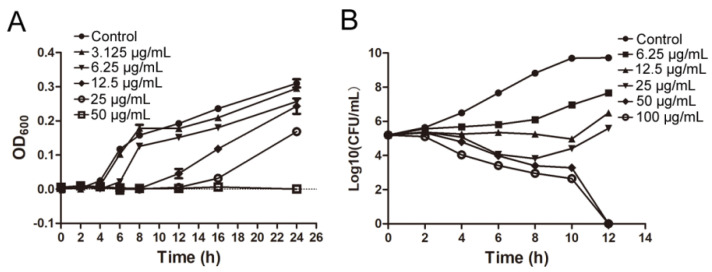
The bacterial growth curve and determination of and bactericidal and bacteriostatic activities of IMB-0042. (**A**) Different concentrations of IMB-0042 (ranging from 3.125 µg/mL to 50 µg/mL) were added to the tubes containing the logarithmic phase cells (diluted to 10^5^ CFU/mL) and incubated at 37 °C with shaking at 150 rpm/min. Samples were collected every hour to measure optical density at 600 nm (OD_600_) to plot the growth curve. (**B**) An initial inoculum of approximately 2 × 10^5^ CFU/mL of *E. coli* ATCC 25,922 was incubated in LB medium containing diffrent concentrations of the compound IMB-0042. The cells were incubated at 37 °C with shaking at 150 rpm/min in the presence of IMB-0042 (ranging from 6.25 µg/mL to 100 µg/mL), and the colony numbers were counted over time.

IMB-0042 could inhibit the growth of *E. coli* ATCC 25,922 with MIC of 25 µg/mL. The time growth curve shows that IMB-0042 inhibits the growth of *E. coli* cells in a dose-dependent manner (Figure 5A). To further understand this inhibitory action of IMB-0042, we used a bactericidal–bacteriostatic curve. As shown in Figure 5B, IMB-0042 showed a bacteriostatic effect at low concentrations (6.25–25 µg/mL), and the number of colonies increased slowly with time, compared with the control group. However, at 50 µg/mL, IMB-0042 showed bactericidal activity, and this activity was increased significantly when the concentration reached 100 µg/mL.

### 3.4. Effect of Compound IMB-0042 on the OM Structure

**Figure 6:**
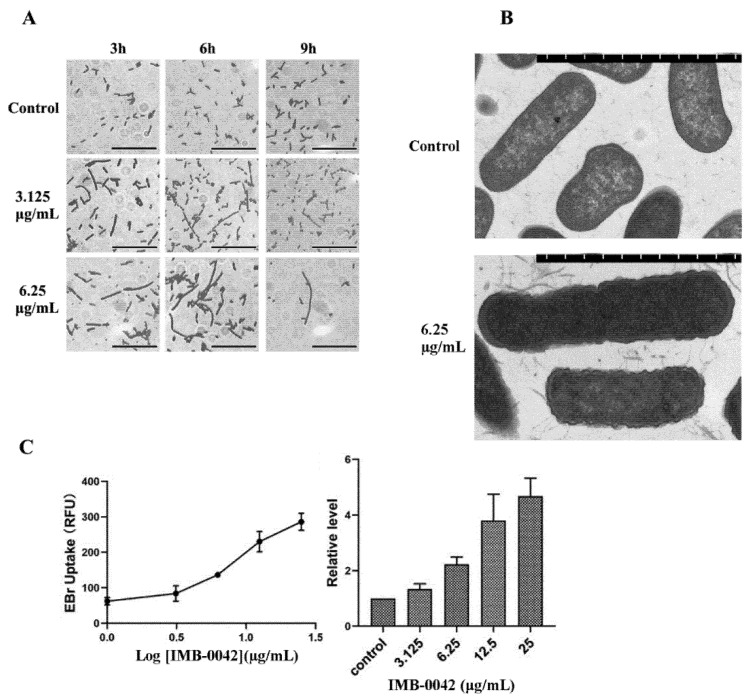
*E. coli* cells were treated with compound IMB-0042 at concentrations of 3.125 µg/mL (1/8 MIC) and 6.25 µg/mL (1/4 MIC) or DMSO, then collected every hour for morphological examination. (**A**) The morphological changes in *E. coli* cells, treated with IMB-0042, were seen under a light microscope. Scale bars, 5 µm. (**B**) The morphological alterations in *E. coli* cells, treated with IMB-0042 for 12 h, were seen with a transmission electron microscope (TEM). Membrane constituents were accumulated, and multilayered membranous bodies were found in the periplasmic space in cells treated with compound IMB-0042. Scale bars, 0.5 µm. (**C**) *E. coli* cells were treated with IMB-0042 (ranging from 3.125µg/mL to 12.5 µg/mL) or DMSO (0.1%) for 12 h. EtBr was then added into the medium to a final concentration of 4 µg/mL. The relative fluorescence intensity is shown.

In *E. coli*, the absence of the Lpt complex causes significant changes in its envelope morphology, resulting in the abnormality of bacterial membrane structure and the accumulation of membrane-forming materials in the periplasmic space [13,14]. It has been reported that Lpt protein-deficient *E. coli* cells show filamentous morphology, which may be related to the failure of cell division [14,19]. If the compound IMB-0042 blocks the interaction between LptA and LptC, the accumulation of LPS in periplasmic space and OM damage should be found in *E. coli* cells. To verify these hypotheses, we cultured *E. coli* with IMB-0042, then observed the cell morphology under an optical microscope and the cell membrane structure with a transmission electron microscope (TEM). As shown in Figure 6A, the cells treated with IMB-0042 became long and filamentous, compared with the control group of cells. The accumulation of membrane constituents and incomplete OM were observed by the TEM (Figure 6B).

EtBr can bind to DNA and fluoresce under UV light. The presence of an intact cell membrane will inhibit the entry of EtBr into the nucleus; indirectly, the intensity of EtBr fluorescence can reflect the damage to the cell membrane [20]. Thus, the intensity of EtBr was measured in IMB-0042 treated *E. coli* cells. The result, shown in Figure 6C, indicated that the EtBr intensity increased in a dose-dependent manner, which showed the damage to OM integrity induced by IMB-0042.

We hypothesized that if IMB-0042 disrupts the interaction between LptA and LptC, a higher level of either LptA or LptC proteins is needed to reduce the antibacterial activity of IMB-0042. To test this hypothesis, we grew *E. coli* BL21 cells, transformed either with the full-length LptA or LptC overexpressing constructs (*pET-16a-lptA* and *pET-16a-lptC*, Figure S2) or with the vector alone (control), then treated with IMB-0042 [15]. The MIC of IMB-0042 with cells harboring the control vector or the LptC overexpressing vector was 6.25 µg/mL; however, the MIC for *E. coli* BL21 containing the LptA overexpressing vector was 12.5 µg/mL. The experiment was repeated four times, and the results were consistent. The results suggest that the strain of overexpressing LptA protein had a reduced sensitivity toward IMB-0042.

The highly selective permeability controlled by the OM prevents many potential antibiotics from entering the cell, which is an important factor that provides inherent drug resistance to Gram-negative bacteria. Because IMB-0042 can perturb the cell envelope and the LPS layer, IMB-0042 may show a synergistic sensitization effect with other antibiotics. We used the checkerboard assay to determine the synergistic effect of IMB-0042 with other antibiotics. As shown in Table 2, IMB-0042 could enhance the efficacy of a variety of antibiotics, such as polymyxin B, Amikacin, and Gentamicin, against *E. coli* ATCC 25922 cells. For some clinical isolates of *E. coli*, IMB-0042 also showed growth-inhibition activity and a synergistic effect with polymyxin B, Amikacin, or Gentamicin (Table S2). In the *mcr*-1 strain, the Polymyxin B resistant strain with *mcr*-1 gene, IMB-0042 can also restore the antimicrobial activity of polymyxin. IMB-0042 also showed growth-inhibition activity against *Klebsiella peneumoniae* (*K. pneumonia*), *Pseudomonas aeruginosa* (*P. aeruginosa*), and *Acinetobacter baumannii* (*A. baumannii*) (MICs were 100 μg/mL, 100 μg/mL, and 25 μg/mL respectively) and synergistic sensitization activity with Polymyxin B. However, for Amikacin and Gentamicin, IMB-0042 only a synergistic effect on *P. aeruginosa* (Table 3).

### 3.5. Toxicity of IMB-0042

We used human renal epithelial cell line 293T to detect the growth inhibition effect of IMB-0042 on human cells. No obvious cytotoxicity was detected when treated with IMB-0042 at 50 µg/mL, indicating that human cells are less sensitive to IMB-0042, compared with *E. coli* cells.

## 4. Discussion

The conditions that can affect the Lpt protein bridge formation, such as Lpt protein depletion or mutations that interfere with protein–protein interactions, can lead to cell growth arrest with an accumulation of newly synthesized LPS in the IM and formation of membranous bodies in the periplasm [14,21,22]. In our previous work, a Y2H model was used to screen compounds that could block the LptA/LptC interaction, and the compound IMB-881 was found to successfully inhibit the LptA/LptC interaction, as well as restricting the growth of *E. coli* [15]. In this study, a similar Y2H model was used, and the compound IMB-0042 was obtained with the capability of blocking LptA/LptC interaction. We performed several assays, including SPR, SEM, and TEM, to confirm our findings. In particular, IMB-0042 showed potent inhibitory activity against the *E. coli* ATCC 25922 strain.

There are many available methods of evaluating protein–protein interaction in vitro, such as isothermal titration calorimetry (ITC), SPR, or biolayer interferometry (BLI). However, these methods need special instruments and materials, and the operation procedure is cumbersome, making it difficult for them to be applied in a high-throughput screening process. In a Y2H system, the blocking of protein–protein interaction can be reflected by the growth inhibition of model bacteria. Therefore, the screening method is simple, economical, and may be used widely in the high-throughput screening of protein–protein interaction inhibitors. However, this screening method has a few drawbacks. First, the fungal cell wall may prohibit the entry of the compounds into the cell, which can lead to false-negative results. Second, compounds that can inhibit the growth of fungus or reduce the expression of the target protein in AH109 cells will also inhibit the growth of AH109(pAD-LptA+pBD-LptC), which can lead to an error hit during the analysis of the results. We adopted some strategies to avoid these problems. During the preliminary screening process, we used a relatively high concentration (100 µg/mL) of the potential drug to permit a high amount of compound to enter the cells. To reduce false positives, AH109(pAD-T+pBD-53) was used as a control. Compared with AH109(pAD-T+pBD-53), the compound with a two-fold decrease MIC on AH109(pAD-LptA+pBD-LptC) was selected. The inhibitory activity was further confirmed by the expression of the *LacZ* reporter gene. In the screening, many compounds showed excellent growth inhibitory activities against both of AH109 strains, but did not show fold differences. Because AH109 is a yeast, this suggests that these compounds may have potential antifungal activity, which is worthy of further study.

The SPR method was used to detect the binding of IMB-0042 to proteins and the blocking of LptA/LptC interactions. When IMB-0042 and the LptA protein flow through the chip at the same time, IMB-0042 also binds with the LptC protein, which is equivalent to increasing the response value and weakening the reduction of the signal value of LptA and LptC that is caused by the blocking effect. We require further methods, other than SPR, to confirm the blocking of LptA/LptC interactions by IMB-0042.

The overexpression of the target protein should reduce the antibacterial activity of antibiotics, but the LptC overexpressing strain showed no obvious change in response to IMB-0042 when compared with the control BL21 (transformed with the vector alone) cells, while LptA overexpression was affected by IMB-0042 and could only show two-fold upregulation. There may be several reasons for this. The BL21 strain is a common host strain to express proteins, and the expressed amount of LptA or LptC are relatively high, which may aggregate together and affect the binding of the IMB-0042 to the protein. In addition, IMB-0042 can bind to both of the proteins, so overexpression of a single protein may lead to weak antagonism, although we cannot rule out the possibility that IMB-0042 may have other intracellular targets. We may need to induce IMB-0042-resistant strains and then analyze the relationship between *LptA* or *LptC* mutations and IMB-0042 resistance.

The bulk of knowledge of the LPS transport has been mainly obtained using *E. coli* as a model system, and the process seems to be structurally conserved among Gram-negative bacteria [23,24,25,26]. IMB-0042 also showed certain growth inhibitory activity against *K. pneumonia*, *P. aeruginosa*, and *A. baumannii*. However, it is unclear whether the disruption of the LptA/LptC protein interaction in these strains has the same lethal effect as that in *E. coli*. We require further research to prove the correlation between the growth inhibitory activity against these strains of IMB-0042 and the disruption of LptA/LptC.

IMB-0042 is a newly discovered compound that can inhibit the growth of *E. coli* ATCC 25922 with MIC 25 µg/mL, but it shows no obvious mammalian cell cytotoxicity, even at 50 µg/mL. In addition, compared with the relatively low antibacterial activity of IMB-0042 itself, IMB-0042 showed good synergistic sensitization activity with Polymyxin B and aminoglycoside compounds, which can greatly reduce the dosage of these drugs in reducing their possible toxicity and overcoming their drug resistance. Therefore, the potential of this drug for use against *E. coli* and other Gram-negative bacterial infection is promising, although further studies and evaluations are needed.

## Figures and Tables

**Table 1:** The kinetics evaluation of the SPR interactions.

Sample	Kon (1/Ms)	Koff (1/s)	KD(M)
LptA with IMB-0042	2.78 × 10^3^	1.30 × 10^−2^	4.67 × 10^−6^
LptC with IMB-0042	1.51 × 10^3^	4.93 × 10^−2^	3.26 × 10^−5^
LptC with LptA	1.69 ×10^5^	3.85 × 10^−1^	2.28 × 10^−6^

**Table 2:** The synergetic effect of IMB-0042 with other antibiotics against *E. coli* ATCC 25922.

Compounds	MIC_Compound_ (μg/mL)		MIC_IMB-0042_ (μg/mL)		FICI
	Single	Combination	Single	Combination	
polymyxin B	0.03	0.00098	25	1.56	0.095
Amikacin	25	0.20	25	0.78	0.039
Gentamicin	12.5	0.05	25	1.56	0.066
Ciprofloxacin	6.25	1.56	25	12.5	0.75
meropenem	0.78	0.39	25	3.13	0.625

**Table 3:** The synergetic effect of IMB-0042 with other antibiotics against clinical isolates of *E. coli* and other Gram-negative bacterial.

Stranis	Compounds	MIC_Compound_ (μg/mL)		MIC_IMB-0042_ (μg/mL)		FICI
		Single	Combination	Single	Combination	
*E. coli* ^a^	polymyxin B	0.1	0.00625		3.13	0.125
	Amikacin	100	6.25	50	3.125	0.125
	Gentamicin	>100	>100		50	>1
*E. coli* ^b^	polymyxin B	0.78	0.2		6.25	0.375
	Amikacin	>100	>100	50	6.25	-
	Gentamicin	>100	>100		50	>1
*E. coli* ^c^	polymyxin B	12.5	6.25		50	1.5
	Amikacin	100	6.25	50	6.25	0.188
	Gentamicin	25	3.13		3.13	0.188
*E. coli* ^d^	polymyxin B	12.5	0.39		1.56	0.094
	Amikacin	6.25	0.39	25	6.25	0.313
	Gentamicin	50	12.5		1.56	0.313
*K. pneumonia*	polymyxin B	6.25	1.56		6.25	0.313
	Amikacin	6.25	6.25	100	50	1.5
	Gentamicin	50	50		50	1.5
*P. aeruginosa*	polymyxin B	25	0.39		3.13	0.047
	Amikacin	>100	0.78	100	0.78	0.0156
	Gentamicin	100	0.39		0.78	0..0117
*A. baumannii*	polymyxin B	6.25	0.024		3.13	0.133
	Amikacin	12.5	6.25	25	12.5	0.75
	Gentamicin	25	6.25		12.5	0.75

^a^^–c^ are clinical isolates, ^d^ is *mcr*-1 strain. *K. peneumoniae* is BAA1706, *P. aeruginosa* is ATCC 27853, *A. baumannii* is ATCC 19606.

## Data Availability

The data presented in this study are available on request from the corresponding author. The gene sequences analyzed in this study can be found in public NCBI Genbank databases.

## Supplementary Materials

The following supporting information can be downloaded at: https://www.mdpi.com/article/10.3390/antibiotics11101385/s1, Figure S1: expression and purification of LptA and LptC. (A) Gene sequence and optimization of GC. (B) Analysis of LptA-induced expression using SDS-PAGE assay. M: Marker; 1: Total cell protein without IPTG induction: 2,4: Cellular lysate supernatant at 20 °C with IPTG induction; 3,5: Inclusion body at 20 °C; 6: Cellular lysate supernatant at 30 °C; 7: Inclusion body at 30 °C. (C) Analysis of LptC-induced expression using SDS-PAGE assay. M: Marker; 1: Total cell protein without IPTG induction; 2: Cellular lysate supernatant at 20 °C with IPTG induction; 3: Inclusion body at 20 °C with IPTG induction; 4: Cellular lysate supernatant at 30 °C with IPTG induction; 5: Inclusion body at 30 °C with IPTG induction; (D,E) Analysis of purified LptA and LptC protein using SDS-PAGE assay. (E,F) Western-blot Analysis of purified LptA and LptC protein with anti-His antibody. Figure S2. Analysis of the overexpression of LptA and LptC. The *pET-16a-**l**ptA* and *pET-16a*-*l**ptC* were constructed and transferred into *E. coli* BL21(DE3) cells to express full length His-tagged LptA and LptC. The expression of the fusion proteins was induced by adding 0.5 mM IPTG into the cell culture and grown overnight at 30 °C. Total proteins in whole cell lysates were analyzed by 12% SDS-PAGE and coomassie blue staining. (A) LptA-induced expression. (B) LptC-induced expression. M: Marker; 1: Total cell protein without IPTG induction; 2; Total cell protein with IPTG induction, Table S1 Plasmids used in this paper, Table S2 Antibiotic sensitivity of clinically resistant strains.
