# Supplementary material for: Identification of a Small Molecule That Inhibits the Interaction of LPS Transporters LptA and LptC
Source: Antibiotics (Basel). 2022 Oct 10;11(10):1385. doi: 10.3390/antibiotics11101385 (PMC9598311; doi:10.3390/antibiotics11101385)
Supplement: Supplementary file 1 [file antibiotics-11-01385-s001.zip › antibiotics-1909329-supplementary.pdf]

antibiotics

MDPI

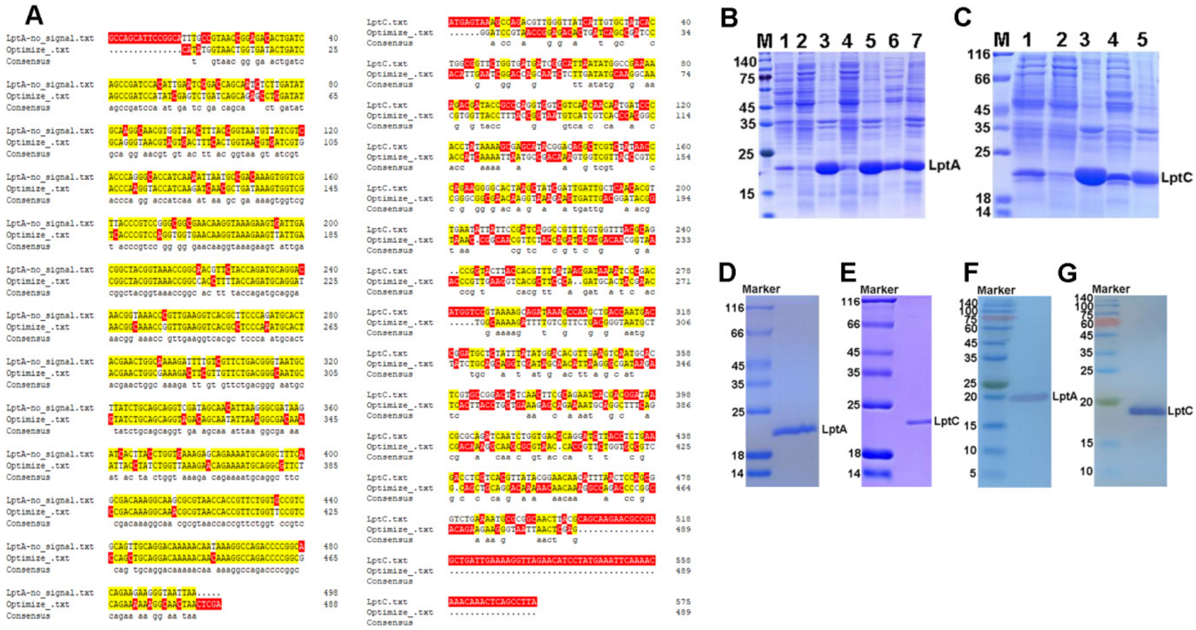

Figure S1.expression and purification of LptA and LptC. (A) Gene sequence and optimization of GC. (B) Analysis of LptA-induced expression using SDS-PAGE assay. M:Marker；1：Total cell protein without IPTG induction；2、4：Cellular lysate supernatant at 20°C with IPTG induction；3、5：Inclusion body at 20°C ；6：Cellular lysate supernatant at 30°C ；7：Inclusion body at 30°C. (C) Analysis of LptC-induced expression using SDS-PAGE assay. M：Marker；1：Total cell protein without IPTG induction；2：Cellular lysate supernatant at 20°C with IPTG induction；3：Inclusion body at 20°C with IPTG induction；4：Cellular lysate supernatant at 30°C with IPTG induction；5：Inclusion body at 30°C with IPTG induction；(D) and (E) Analysis of purified LptA and LptC protein using SDS-PAGE assay. (E) and (F) Western-blot Analysis of purified LptA and LptC protein with anti-His antibody.

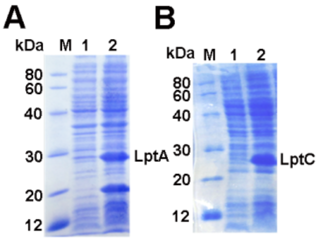

Figure S2. Analysis of the overexpression of LptA and LptC. The *pET-16a-lptA* and *pET-16a-lptC* were constructed and transferred into *E. coli* BL21(DE3) cells to express full length His-tagged LptA and LptC. The expression of the fusion proteins was induced by adding 0.5 mM IPTG into the cell culture and grown overnight at 30°C. Total proteins in whole cell lysates were analyzed by 12% SDS-PAGE and coomassie blue staining. (A) LptA-induced expression. (B) LptC-induced expression. M: Marker; 1：Total cell protein without IPTG induction；2；Total cell protein with IPTG induction

Table S1 Plasmids used in this paper

name	Description	Reference
pAD-lptA	Expression vector for LptA -AD-HA	Xuelian Zhang,et al.,2019
pBD-lptC	Expression vector for LptC-DNA-BD-Myc	Xuelian Zhang,et al.,2019
pAD-t	Expression vector for T-AD-Myc	Xuelian Zhang,et al.,2019
pBD-53	Expression vector for 53-DNA-BD-Myc	Xuelian Zhang,et al.,2019
pET-28a-lptA	Expression vector for LptA-His_6 (28-185)	In this study
pET-28a-lptC	Expression vector for LptC-His_6(24-191)	In this study
pET-16a-lptA	Expression vector for LptA-His_6	Xuelian Zhang,et al.,2019
pET-16a-lptC	Expression vector for LptC-His_6	Xuelian Zhang,et al.,2019

Table S2 Antibiotic sensitivity of clinically resistant strains

	MIC (μg/mL)			
Antibiotics	*E.coli*[a]	*E.coli*[b]	*E.coli*[c]	*E.coli*[d]
Polymycin B	<0.5	⩽1	>1	>1
Amikacin	>64	>64	>64	-
Gentamicin	>64	>64	>8	-
Ceftazidime	>16	>16	>16	-
Meropenem	⩽1	>8	8	-
Cefotaxime	>32	>16	2	-
Levofloxacin	>8	>8	4	-
Cyclopropane	>2	>2	1	-
Ampicillin	>16	>32	⩽2	-
cefatriaxone	R	R	R	-
Source	blood	sputum	urine	feces

E.coli[a-d] are four strains used in Table S2. a-c are isolates from clinical patients and d is isolate with *mcr-1* gene from goat feces in the farm.
